# Integrating sarcopenia and non-contrast CT radiomics for preoperative prediction of survival in sarcomatoid renal cell carcinoma

**DOI:** 10.3389/fonc.2025.1637032

**Published:** 2025-10-22

**Authors:** Tongpeng Liu, Zhijian Zhou, Yu Yao, Yang Hu, Lijiang Sun, Guiming Zhang

**Affiliations:** ^1^ Department of Urology, The Affiliated Hospital of Qingdao University, Qingdao, China; ^2^ Department of Radiology, Peking University People’s Hospital Qingdao Hospital, Qingdao, China

**Keywords:** sarcomatoid renal cell carcinoma, sarcopenia, radiomics, prognosis, non-contrast CT, survival analysis, machine learning, preoperative prediction

## Abstract

**Background:**

Sarcomatoid renal cell carcinoma (sRCC) is an aggressive subtype with a poor prognosis. Preoperative prognostic tools are lacking, and the predictive value of sarcopenia combined with radiomic features from non-contrast CT remains unexplored.

**Methods:**

In this retrospective study, 121 pathologically confirmed sRCC patients were enrolled. Sarcopenia was assessed using muscle mass measurements at the L3 level on preoperative non-contrast CT. Radiomic features were extracted from tumor regions of interest. Least absolute shrinkage and selection operator (LASSO) and Cox regression were used to select features and construct prognostic models for overall survival (OS). A combined model integrating sarcopenia status and radiomic signature (Rad-score) was developed and evaluated regarding its discrimination, calibration, and clinical utility.

**Results:**

Multivariable analysis identified paravertebral muscle-defined sarcopenia (HR = 3.046, p = 0.029), platelet-to-neutrophil ratio, hemoglobin-albumin-lymphocyte-platelet score, tumor size, and N stage as independent prognostic factors. The combined model (clinical + Rad-score) demonstrated superior predictive performance for 1-, 2-, and 3-year OS, with AUCs of 0.849, 0.804, and 0.819, respectively, and significantly outperformed the radiomics-only model (p = 0.002). Calibration curves and decision curve analysis confirmed its clinical applicability.

**Conclusion:**

The integration of sarcopenia and non-contrast CT radiomics provides a valuable preoperative tool for predicting survival in sRCC patients, facilitating individualized risk stratification and clinical decision-making.

## Introduction

Sarcomatoid renal cell carcinoma (sRCC) is a rare and highly aggressive subtype of renal cell carcinoma characterized by sarcomatoid differentiation, accounting for approximately 4–5% of all RCC cases (1–4). Patients with sRCC often present with advanced disease and suffer from a dismal prognosis, with a five-year survival rate significantly lower than that of other renal cell carcinoma (RCC) subtypes (3). Although surgical resection remains the primary treatment for localized sRCC, the risk of postoperative recurrence and metastasis is substantial, and median overall survival is typically less than 12 months (5–7). Thus, the early identification of patients at high risk of recurrence or mortality is of critical clinical importance for developing individualized treatment strategies.

Current prognostic assessment of sRCC relies predominantly on postoperative pathological features, such as the proportion of sarcomatoid component, tumor stage, and Ki-67 index (8, 9). However, these indicators require surgical specimens, precluding preoperative risk evaluation and limiting opportunities for early intervention. Furthermore, the relatively low response rates of sRCC to conventional targeted therapies and immunotherapy underscore the urgent need for developing preoperative prognostic biomarkers (10, 11).

Sarcopenia is frequently observed in patients with advanced RCC, particularly those with high tumor burden or vascular invasion, and often coexists with cachexia (12). It is significantly associated with increased postoperative complications, reduced tolerance to chemotherapy, and shortened overall survival (13). The underlying mechanisms may involve systemic inflammation, dysregulated protein metabolism, and immune suppression (14). Nevertheless, the prognostic value of sarcopenia in sRCC patients remains incompletely understood.

In recent years, machine learning (ML) algorithms have gained considerable attention in medical research due to their capability to integrate multi-source data and construct high-dimensional predictive models (15). Radiomics has emerged as a promising approach for non-invasively decoding tumor heterogeneity by extracting high-dimensional quantitative features from standard medical images, thereby predicting tumor biological behavior (16, 17). Non-contrast CT, widely used in renal cancer diagnostics, offers broad availability and standardization, and its radiomic features have demonstrated potential in distinguishing RCC subtypes, predicting tumor grade, and assessing prognosis (15, 18–20). However, no study to date has integrated pretreatment sarcopenia with radiomic features from non-contrast CT for predicting postoperative survival in sRCC patients.

Based on this background, we hypothesize that preoperative sarcopenia combined with radiomic features from non-contrast CT may collectively influence postoperative survival in sRCC. This study aims to investigate the potential of sarcopenia as a preoperative predictor and to evaluate whether its integration with radiomic features can enhance the accuracy of survival prediction, thereby providing an imaging-based foundation for preoperative risk stratification and individualized therapeutic decision-making. 

## Materials and methods

### Study design and participants

This retrospective cohort analysis included patients pathologically diagnosed with sRCC at our institution between December 2009 and September 2024. The study protocol was approved by the Ethics Committee of The Affiliated Hospital of Qingdao University (Approval No: QYFYWZLL30031) and conducted in accordance with the ethical principles of the Declaration of Helsinki (2013 revision). Informed consent was waived due to the retrospective nature of the study. Clinical data were independently and blindly collected by two researchers. Inclusion criteria were: (1) postoperative pathological confirmation of sRCC with complete clinical records; (2) abdominal CT scan performed within one month before surgery. Exclusion criteria were: (1) incomplete clinical, pathological, or follow-up data; (2) concurrent other malignancies or multi-organ dysfunction; (3) previous neoadjuvant therapy; (4) absence of DICOM-format CT images meeting quality standards; (5) death due to complications within 30 days after surgery; (6) active infection or recent use of anti-inflammatory/immunosuppressive drugs. The study flowchart is shown in **Supplementary Figure 1**. Clinical variables included age, blood biochemical indicators, and pathological characteristics. Missing values (<5%) were handled using multiple imputation.

### Follow-up and endpoints

A standardized postoperative follow-up protocol was implemented: assessments every 3–4 months in the first year, every 6 months from years 2 to 5, and annually thereafter. Evaluations included clinical symptoms, laboratory tests (e.g., complete blood count and biochemistry), and imaging (CT or MRI). Follow-up concluded on April 1, 2025. The primary endpoint was overall survival (OS), defined as the duration from pathological diagnosis to death from any cause or the last confirmed follow-up.

### CT image acquisition

Preoperative non-contrast CT images were obtained using multiple scanners: GE Optima CT620, LightSpeed CT750 HD, Optima CT670, Revolution CT (GE Healthcare, USA), and Siemens SOMATOM Sensation64 and Definition Flash (Siemens Healthineers, Germany). Scanning parameters were: tube current 240–320 mAs (automatically modulated), voltage 120 kVp, pitch 1.375, reconstruction matrix 512×512, and slice thickness 5 mm. All images were exported in DICOM format from the PACS for further processing.

### Tumor segmentation and radiomic feature extraction

Tumor segmentation and feature extraction were performed using a standardized protocol. One radiologist (7 years of abdominal imaging experience) and one urologist (15 years of urologic oncology experience), both blinded to pathology, manually delineated tumor boundaries on non-contrast CT images slice-by-layer using ITK-SNAP (v4.2.0) to generate 3D regions of interest (ROIs), carefully excluding adjacent renal parenchyma and perinephric fat. Discrepancies were resolved by a third urologist with 35 years of experience. Prior to feature extraction, all images underwent standardized preprocessing including resampling and gray-level discretization. Features were extracted in Python 3.7 using the pyradiomics toolbox, following the Image Biomarker Standardisation Initiative (IBSI) guidelines (21). Extracted features included first-order statistics, shape, gray-level co-occurrence matrix (GLCM), gray-level dependence matrix (GLDM), gray-level run-length matrix (GLRLM), gray-level size zone matrix (GLSZM), neighboring gray-tone difference matrix (NGTDM), and wavelet-derived features. To evaluate segmentation reproducibility, two blinded urologists independently segmented ROIs on 30 randomly selected CT images. The first reader repeated the segmentation after one month for intra-observer consistency assessment. Features with an intraclass correlation coefficient (ICC) > 0.75 were retained for further analysis.

### Body composition assessment and sarcopenia diagnosis

**Figure 1:**
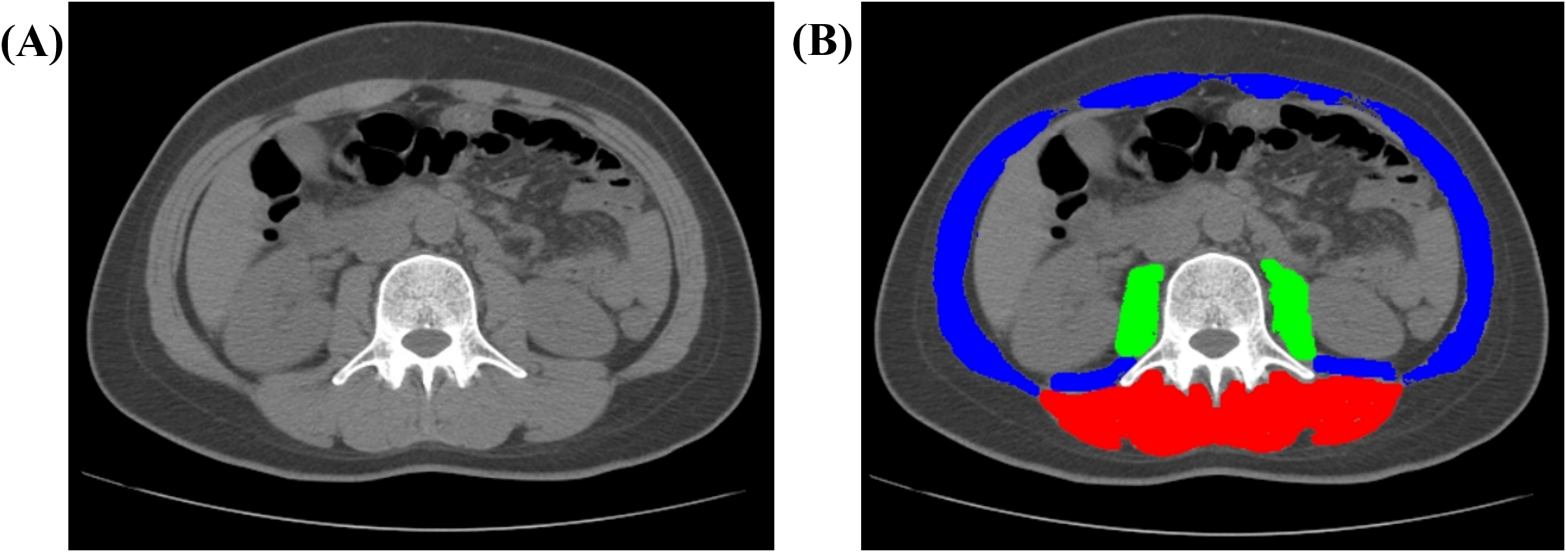
Representative CT image analysis of a 41-year-old female patient. **(A)** Axial non-contrast CT image acquired at the level of the third lumbar vertebra (L3). **(B)** Automated segmentation of muscle compartments: paraspinal muscles (PS, red), psoas major muscles (PM, green), and total abdominal muscle (TAM, combined overlay). Abbreviations: CT, computed tomography; L3, third lumbar vertebra; TAM, total abdominal muscle; PM, psoas major; PS, paraspinal muscles.

Body composition was quantified at baseline using CT axial images at the third lumbar (L3) level. SliceOmatic 5.0 (Tomovision, Canada) was used to measure cross-sectional areas (cm²) of total abdominal muscle (TAM), psoas muscle (PM), and paraspinal muscles (PS). Muscle tissue was defined using Hounsfield unit (HU) thresholds (−29 to 150 HU) (22), with manual correction for accuracy, as illustrated in **Figure 1**. All analyses were performed by one radiologist with 7 years of experience. Height-adjusted indices (TAM/height², PM/height²) were derived (23, 24). Sarcopenia was defined using established criteria (23–25): height-adjusted TAM index <52.4 cm²/m² (men) or <38.5 cm²/m² (women); PM index <6.36 cm²/m² (men) or <3.92 cm²/m² (women); absolute PS area <31.97 cm² (men) or <28.95 cm² (women).

### Feature selection and radiomics model construction

Radiomic features were Z-score normalized. A multi-stage selection strategy was applied: first, features with ICC > 0.75 were retained; second, low-variance features (variance threshold <0.1) were removed, and highly correlated features (|r| > 0.9) were reduced by retaining those with higher variance. Univariate Cox regression (p < 0.001) identified prognosis-related features, followed by Least absolute shrinkage and selection operator (LASSO) regression with 10-fold cross-validation (optimal λ selected via minimum error) for dimensionality reduction. Five machine learning algorithms (SuperPC, stepwise Cox, random survival forest [RSF], CoxBoost, and plsRcox) were used to build prognostic models. The model with the highest C-index was selected to generate a radiomics score (Rad-score) for subsequent analysis.

### Clinical and combined model construction

Three models were developed and compared. Univariate Cox regression (p < 0.05) identified potential prognostic variables, followed by LASSO regression (10-fold cross-validation, λ.min) for variable selection. Multivariable Cox regression with backward likelihood ratio (LR) method identified independent prognostic factors to build a clinical model centered on sarcopenia. A combined model was constructed by integrating the Rad-score and significant clinical variables via multivariable Cox regression. Model performance was compared among the radiomics, clinical, and combined models following TRIPOD guidelines.

### Model evaluation and interpretation

Internal validation included repeated 10-fold cross-validation for C-index calculation and bootstrap resampling (1000 repetitions) for confidence intervals. Delong’s test and bootstrap methods (1000 repetitions) were used to compare C-indices between models. A nomogram based on the combined model was developed to predict survival probabilities. Time-dependent ROC curves assessed discrimination at 1, 2, and 3 years. Calibration curves (1000 bootstrap samples) evaluated agreement between predicted and observed outcomes. Decision curve analysis (DCA) quantified clinical utility by calculating net benefit across threshold probabilities. SHAP (Shapley Additive exPlanations) analysis interpreted feature contributions and enhanced model transparency (26, 27).

### Statistical analysis

All analyses were performed using IBM SPSS Statistics 26.0 and R 4.4.3. Two-sided p-values < 0.05 were considered statistically significant. Categorical variables are presented as counts and percentages, compared using Pearson’s χ² or Fisher’s exact test. Continuous variables were tested for normality using Shapiro–Wilk test; normally distributed variables are expressed as mean ± standard deviation and compared with t-tests, while non-normal variables are reported as median (IQR) and compared with Mann–Whitney U test.

## Results

### Baseline clinical characteristics

This retrospective cohort study strictly adhered to predefined inclusion and exclusion criteria, ultimately enrolling 121 patients with pathologically confirmed sRCC. All patients received standardized treatment and systematic follow-up. The median follow-up time for the entire cohort was 21 months (range: 1–183 months). By the end of follow-up, 60 deaths had been recorded. Detailed baseline characteristics, including demographic, clinical, and pathological parameters, are summarized in **Table 1**.

**Table 1:** Baseline demographic, clinical, and pathological characteristics of the 121 patients with sarcomatoid renal cell carcinoma (sRCC) included in the retrospective cohort study.

Characteristic	Category	Value (mean ± SD, median [IQR], or number [%])
Age, years		57.26 ± 12.28
Sex	Male	86(71.10)
	Female	35(28.90)
BMI		24.21 ± 3.59
TAM index		43.74 ± 7.97
PMI		4.91 ± 1.44
PS		43.96 ± 10.23
Hypertension	Yes	50(41.30)
	No	71(58.70)
Diabetes	Yes	23(19.00)
	No	98(81.00)
Albumin		38.88 ± 6.63
Alkaline Phosphatase		101.54 ± 69.04
Cholesterol		4.40 ± 1.16
LDH		216.16 ± 149.96
Urea		5.54 ± 1.95
Creatinine		83.98 ± 64.83
Glucose		6.20 ± 2.50
Fibrinogen		4.72 ± 1.43
T stage	T1/T2	55(45.50)
	T3/T4	66(54.50)
N stage	N0	81(66.90)
	N1	40(33.10)
M stage	M0	78(64.50)
	M1	43(35.50)
Tumor Size		7.59 ± 3.36
Ki-67 Index		0.30 ± 0.21
SII		1176.34 ± 1025.56
PLR		210.42 ± 117.09
LMR		3.16 ± 1.99
PNR		68.27 ± 30.88
PAR		8.38 ± 3.83
GLR		4.40 ± 3.01
PNI		47.33 ± 7.84
HALP		30.23 ± 18.31
TAM-defined sarcopenia	Yes	88(72.70)
	No	33(27.30)
PM-defined sarcopenia	Yes	88(72.70)
	No	33(27.30)
PS-defined sarcopenia	Yes	6(5.00)
	No	115(95.00)
Characteristic		Value (mean ± SD, median [IQR], or number [%])
OS, months		21(8.50-50.00)
State of life	Survival	61(50.40)
	Death	60(49.60)

SD, Standard deviation; IQR, Interquartile range; BMI, Body mass index; TAM, Total abdominal muscle; PM, Psoas muscle; PS, Paraspinal muscle; PMI, Paraspinal muscle index; LDH, Lactate dehydrogenase; SII, Systemic immune-inflammation index; PLR, Platelet-to-lymphocyte ratio; LMR, Lymphocyte-to-monocyte ratio; PNR, Platelet-to-neutrophil ratio; PAR, Platelet-to-albumin ratio; GLR, Glucose-to-lymphocyte ratio; PNI, Prognostic nutritional index; HALP, Hemoglobin, albumin, lymphocyte, and platelet index.

### Radiomic feature selection, prognostic model construction, and interpretation

**Figure 2:**
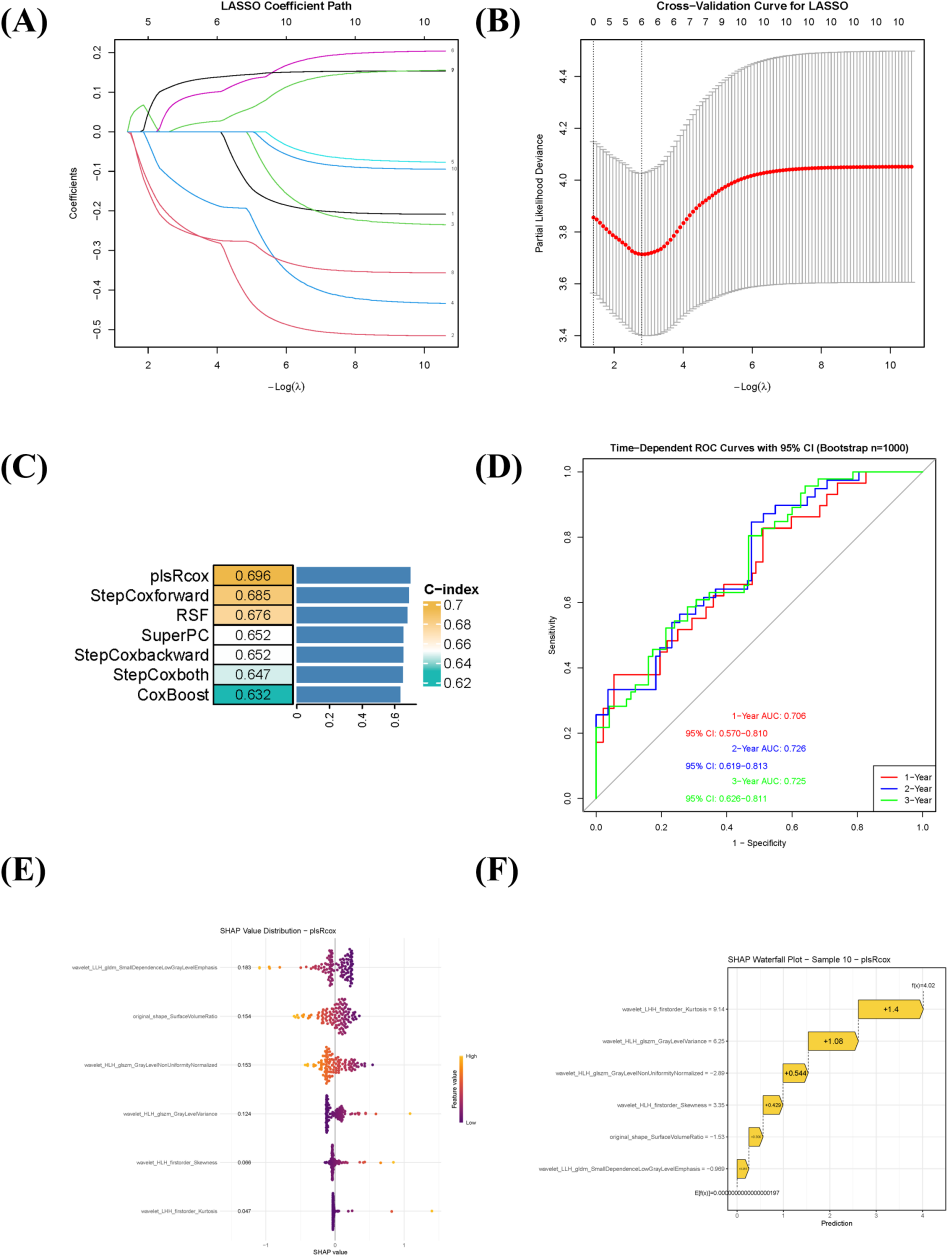
Radiomics feature selection, prognostic model construction, and interpretation using SHAP. **(A)** LASSO coefficient profile of the 10 radiomic features significantly associated with overall survival (univariate Cox regression, P < 0.001). **(B)** Ten-fold cross-validation curve for tuning parameter (λ) selection in the LASSO regression. The optimal λ value is indicated by the vertical dotted line. **(C)** Comparison of six prognostic modeling approaches based on the C-index. The plsRcox model showed the highest predictive performance (C-index = 0.696). **(D)** Time-dependent receiver operating characteristic (ROC) curves of the plsRcox model for predicting 1-, 2-, and 3-year overall survival. **(E)** Global SHAP summary plot displaying the magnitude and direction of the influence of the six selected radiomic features on the model output. All features contributed positively to risk prediction. **(F)** SHAP waterfall chart illustrating the decomposition of the predicted risk score for an individual high-risk patient, showing the additive contribution of each feature relative to the baseline prediction.

A total of 854 quantitative radiomic features were extracted from the ROIs. After evaluating intra- and inter-observer consistency (ICC > 0.75), 707 features were retained for further analysis. Subsequent low-variance filtering (variance threshold < 0.1) and removal of highly correlated features (retaining those with higher variance in each correlated group) yielded 186 features. Univariate Cox regression identified 10 features significantly associated with prognosis (P < 0.001). LASSO regression was then applied for further dimensionality reduction, resulting in six highly predictive features for model construction (**Figures 2A, B**). To comprehensively evaluate predictive performance, five algorithmic strategies were systematically compared. The plsRcox model demonstrated optimal performance (**Figure 2C**), achieving a C-index of 0.696 via 10-fold cross-validation. Time-dependent ROC analysis showed that the model yielded AUC values of 0.706, 0.726, and 0.725 for predicting 1-, 2-, and 3-year OS, respectively (**Figure 2D**). SHAP analysis was used to interpret the plsRcox model. The global SHAP summary plot (**Figure 2E**) illustrated the direction and magnitude of contributions of the six key features, all of which acted as positive predictors. An individual prediction analysis (**Figure 2F**) deconstructed the prediction for a high-risk patient: the baseline prediction (E[f(x)] = −1.97×10^-17^) represents the model’s output reference, while the individual prediction value (f(x) = 4.02) indicated elevated mortality risk.

### Prognostic factor selection and combined model construction

Univariate Cox regression identified clinical features significantly associated with OS in sRCC patients (**Table 2**). LASSO regression was used to screen prognostic variables, including PNR, PAR, HALP, Ki-67 index, tumor size, N stage, M stage, sarcopenia defined by TAM index, and sarcopenia defined by PS area (**Supplementary Figure 2**). Subsequent multivariate Cox regression using the backward likelihood ratio method identified the following independent prognostic factors for OS: PNR (HR = 0.981, 95% CI: 0.971–0.991; P < 0.001), HALP (HR = 0.979, 95% CI: 0.963–0.995; P = 0.01), tumor size (HR = 1.074, 95% CI: 0.996–1.157; P = 0.064), N stage (HR = 2.434, 95% CI: 1.387–4.270; P = 0.002), and PS-defined sarcopenia (HR = 3.046, 95% CI: 1.119–8.289; P = 0.029). A clinical prognostic model based on these variables was constructed, and a clinical risk score was computed for each patient. Finally, a combined prognostic model was established by integrating the radiomics risk score (Rad-score) with the clinical model.

**Table 2:** Univariate and multivariate Cox regression analyses of factors associated with overall survival in patients with sarcomatoid renal cell carcinoma (sRCC).

Variable	Univariate analysis			Multivariate analysis		
	HR	95% CI	P value	HR	95% CI	P value
Age	0.996	0.977-1.016	0.707			
Gender						
Female	Ref					
Male	0.917	0.531-1.584	0.757			
BMI	0.927	0.868-0.991	<0.026			
Hypertension						
No	Ref					
Yes	0.612	0.357-1.049	0.074			
Diabetes						
No	Ref					
Yes	0.750	0.369-1.528	0.429			
Albumin	0.956	0.919-0.995	0.029			
Alkaline Phosphatase	1.002	0.999-1.006	0.153			
Cholesterol	0.997	0.793-1.254	0.979			
LDH	1.002	1.000-1.003	0.022			
Urea	0.885	0.768-1.019	0.089			
Creatinine	1.000	0.997-1.003	0.919			
Glucose	1.051	0.940-1.176	0.384			
Fibrinogen	1.213	1.031-1.427	0.020			
Tumor Size	1.105	1.033-1.182	0.003	1.074	0.996-1.157	0.064
Ki-67	6.215	1.963-19.678	0.002			
T stage						
T1/T2	Ref					
T3/T4	2.060	1.202-3.530	0.009			
N stage						
N0	Ref			Ref		
N1	2.413	1.443-4.034	0.001	2.434	1.387-4.270	0.002
M stage						
M0	Ref					
M1	2.428	1.458-4.044	0.001			
SII	1.000	1.000-1.000	0.003			
PLR	1.002	1.000-1.004	0.025			
LMR	0.704	0.563-0.879	0.002			
PNR	0.990	0.979-1.000	0.052	0.981	0.971-0.991	<0.001
PAR	1.083	1.021-1.150	0.008			
GLR	1.049	0.969-1.135	0.239			
PNI	0.960	0.930-0.991	0.013			
HALP	0.974	0.958-0.990	0.001	0.979	0.963-0.995	0.010
TAM-defined sarcopenia						
No	Ref					
Yes	2.002	1.059-3.784	0.033			
PM-defined sarcopenia						
No	Ref					
Yes	1.284	0.721-2.888	0.395			
PS-defined sarcopenia						
No	Ref			Ref		
Yes	2.804	1.112-7.071	0.029	3.046	1.119-8.289	0.029

BMI, Body mass index; TAM, Total abdominal muscle; PM, Psoas muscle; PS, Paraspinal muscle; LDH, Lactate dehydrogenase; SII, Systemic immune-inflammation index; PLR, Platelet-to-lymphocyte ratio; LMR, Lymphocyte-to-monocyte ratio; PNR, Platelet-to-neutrophil ratio; PAR, Platelet-to-albumin ratio; GLR, Glucose-to-lymphocyte ratio; PNI, Prognostic nutritional index; HALP, Hemoglobin, albumin, lymphocyte, and platelet index.

### Prognostic model based on sarcopenia and radiomics and its interpretation

**Figure 3:**
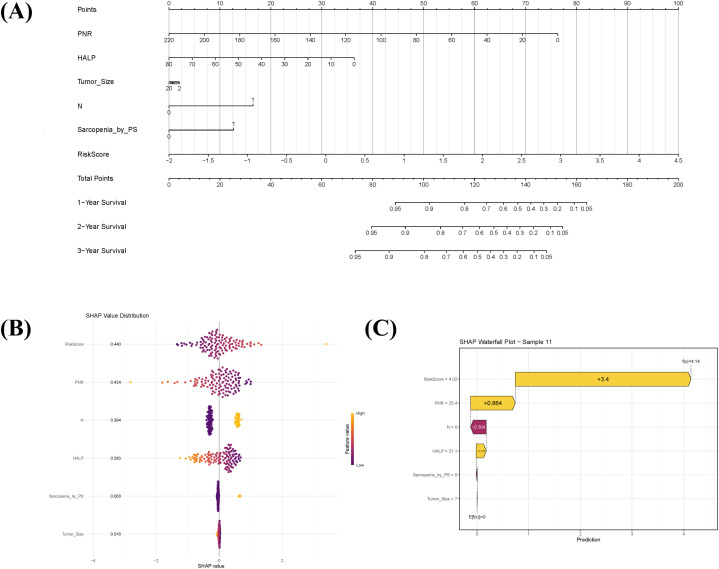
Prognostic nomogram integrating sarcopenia-associated clinical factors and radiomic features, with model interpretability analysis. **(A)** Nomogram for predicting 1-, 2-, and 3-year overall survival in sarcomatoid renal cell carcinoma (sRCC) patients, combining sarcopenia-related clinical predictors with the radiomics score (Radscore). To apply the nomogram: assign points for each variable on the top axis, sum the points, and locate the total on the survival probability axes to estimate predicted survival. **(B)** SHAP summary plot illustrating the overall contribution of each predictor in the combined model. Each dot represents an individual patient; positive SHAP values indicate variables associated with increased mortality risk. **(C)** SHAP waterfall chart explaining the prediction for a representative high-risk patient (Sample 11). The baseline prediction (E[f(x)]=0) reflects the average population risk, while the patient’s elevated risk score (f(x)=4.14) is primarily driven by high Radscore and PNR.

Using the selected clinical prognostic factors and the Rad-score, a nomogram was developed to predict 1-, 2-, and 3-year OS in sRCC patients (**Figure 3A**). The nomogram is applied as follows: (1) determine the points for each variable on the top point scale; (2) project each point vertically to the “Points” axis; (3) sum all points to obtain the total score; (4) determine the corresponding 1-, 2-, and 3-year survival probabilities on the bottom survival probability axis. SHAP analysis was further employed to interpret the combined model. The global SHAP beeswarm plot (**Figure 3B**) revealed that all four key predictive features exhibited positive contributions (SHAP values > 0), indicating significant associations with poor prognosis. Individual prediction visualization (**Figure 3C**) illustrated an example of a high-risk patient: the baseline prediction (E[f(x)] = 0) represents the model’s risk reference, while the individual prediction (f(x) = 4.14) was substantially higher, consistent with actual high-risk clinical outcomes.

### Predictive performance and clinical validation of the combined prognostic model

The combined model demonstrated superior discriminative ability for predicting OS in sRCC patients compared to the sarcopenia-based clinical model and the radiomics model alone. Based on repeated cross-validation, the mean C-indices for the clinical, radiomics, and combined models in the training cohort were 0.746, 0.696, and 0.783, respectively. Pairwise comparisons using Delong’s test indicated a statistically significant difference between the combined model and the radiomics model (p = 0.002), while differences between the clinical and radiomics models (p = 0.081) and between the clinical and combined models (p = 0.216) were not statistically significant.

**Figure 4:**
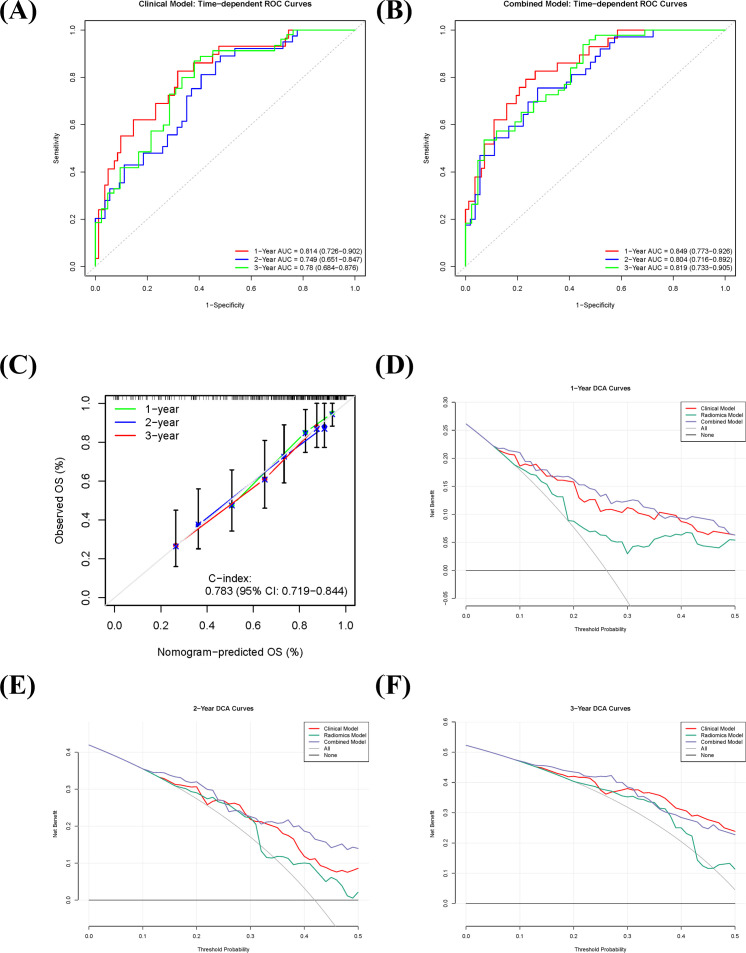
Predictive performance and clinical utility of the combined prognostic model in sarcomatoid renal cell carcinoma (sRCC). **(A, B)** Time-dependent receiver operating characteristic (ROC) curves of the clinical model **(A)** and the combined model **(B)** for predicting 1-, 2-, and 3-year overall survival. **(C)** Calibration curve of the combined model, showing the agreement between predicted and observed survival probabilities. **(D–F)** Decision curve analysis (DCA) for 1-year **(D)**, 2-year **(E)**, and 3-year **(F)** overall survival, comparing the net benefit of the combined model against the clinical model, radiomics model, and extreme intervention strategies.

Time-dependent ROC analysis further validated the predictive accuracy of the models. The sarcopenia-based clinical model achieved AUC values of 0.814 (95% CI: 0.726–0.902), 0.749 (95% CI: 0.651–0.847), and 0.780 (95% CI: 0.684–0.876) for predicting 1-, 2-, and 3-year OS, respectively (**Figure 4A**). The corresponding AUC values for the combined model were 0.849 (95% CI: 0.773–0.926), 0.804 (95% CI: 0.725–0.883), and 0.819 (95% CI: 0.733–0.905) (**Figure 4B**). Calibration curves showed good agreement between predicted and observed survival probabilities for the combined model (**Figure 4C**). DCA indicated that the combined model offered high clinical utility across most threshold probabilities for 1-, 2-, and 3-year survival predictions, with net benefit exceeding those of the “treat-all” and “treat-none” strategies (**Figures 4D–F**), supporting its potential for clinical application.

## Discussion

This study is the first to integrate pretreatment sarcopenia with radiomic features from non-contrast CT to develop and validate a combined model for predicting postoperative OS in patients with sRCC. The combined model demonstrated good discriminative ability for predicting 1-, 2-, and 3-year OS, with AUC values of 0.849, 0.804, and 0.819, respectively. It significantly outperformed the radiomics-only model (p = 0.002) and consistently showed higher C-indices and AUCs compared to the clinical-only model, although this difference did not reach statistical significance (p = 0.216). Furthermore, the combined model exhibited good calibration and clinical utility in decision curve analysis, supporting its potential value in individualized prognostic assessment.

Sarcopenia, an important indicator of nutritional and inflammatory status, was identified in this study as an independent prognostic factor in sRCC. Specifically, sarcopenia defined by PS area was significantly associated with poorer outcomes (HR = 3.046, p = 0.029), consistent with previous studies in clear cell renal cell carcinoma and other solid tumors (28–31). The underlying mechanisms are multifactorial, involving not only classic inflammatory pathways and protein metabolism dysregulation, but also gut microbiota dysbiosis, immunosenescence, and chronic oxidative stress, together forming a complex pathological network (32–34). Age-related gut dysbiosis is characterized by a reduction in beneficial bacteria (e.g., Bacteroides, Bifidobacterium, and short-chain fatty acid [SCFA]-producing bacteria) and an increase in opportunistic pathogens (e.g., Proteobacteria) (35–37). These changes lead to decreased production of SCFAs such as butyrate (38, 39), impair intestinal barrier integrity, and promote translocation of microbial-associated molecular patterns (MAMPs) into the circulation, triggering a systemic low-grade inflammatory state (40, 41). Inflammatory cytokines (e.g., TNF-α, IL-6) activate NF-κB and MAPK signaling pathways, exacerb muscle protein degradation and suppressing synthesis, thereby directly promoting sarcopenia (42, 43). From a redox perspective, sarcopenia is closely linked to chronic oxidative stress. Under physiological conditions, reactive oxygen species (ROS) and reactive nitrogen species (RNS) contribute to muscle adaptation and regeneration; however, under pathological conditions such as malignancy, aging, or chronic inflammation, excessive ROS/RNS production induces oxidative stress, leading to mitochondrial dysfunction, protein oxidation, lipid peroxidation, and DNA damage. These processes promote protein degradation, inhibit synthesis, and induce apoptosis and necrosis of muscle cells (44, 45). Moreover, accumulation of advanced glycation end-products (AGEs) and advanced lipoxidation end-products (ALEs) can cause muscle protein cross-linking and functional loss, and exacerbate atrophy through activation of RAGE-mediated inflammatory pathways such as NF-κB (46, 47). Our multivariate analysis also confirmed the prognostic value of clinical indicators including PNR, HALP, tumor size, and N stage, enriching the prognostic toolkit for sRCC and supporting potential applications in perioperative management, treatment strategy discussion, and personalized follow-up planning.

In terms of radiomics, six features significantly associated with OS were selected from non-contrast CT images to construct a radiomic model with a C-index of 0.696, indicating moderate predictive ability. However, the limited performance of radiomics alone suggests that imaging features may not fully capture the high heterogeneity and complex biology of sRCC. Notably, SHAP analysis revealed that all selected radiomic features were positive predictors, collectively indicating poorer prognosis, possibly related to intratumoral necrosis, fibrosis, or microenvironment dysregulation. The combined model integrating clinical and radiomic features allowed complementary multi-dimensional risk assessment and significantly improved the identification of high-risk patients. Previous studies have shown that radiomic features can effectively reflect tumor heterogeneity, microenvironment, and biological behavior, providing non-invasive quantitative information closely related to pathological characteristics. Multiple studies have successfully developed radiomic models based on CT, MRI, and PET/CT to predict ISUP grade, metastatic potential, and prognosis in RCC, demonstrating considerable clinical value (48–50). Importantly, radiomics has shown promise not only in tumor grading but also in prognostic stratification. Zhao et al. (51) developed a model based on intravoxel incoherent motion (IVIM) diffusion-weighted imaging for preoperative prediction of nuclear grade and survival in ccRCC with venous tumor thrombus, outperforming conventional imaging metrics. Other studies have combined radiomics with existing clinical scoring systems (e.g., SSIGN score, Leibovich score) to improve prognostic accuracy (52–54). For example, Li et al. (54) validated a CT-based deep learning radiomic model for Leibovich risk stratification in non-metastatic ccRCC across multiple centers, suggesting its utility as a complement to existing clinical tools. The significant difference between the combined and radiomics-only models (p = 0.002) underscores the contribution of clinical variables such as sarcopenia. Although the difference between the combined and clinical-only models was not statistically significant (p = 0.216), the consistent advantage in time-dependent ROC analysis and C-index suggests more stable predictive performance of the integrated model.

This study has several limitations. First, its retrospective single-center design and relatively small sample size may introduce selection bias. Second, although consistency was assessed, manual ROI delineation is subject to subjective variability; future studies could employ deep learning-based auto-segmentation to improve reproducibility and efficiency. Third, dynamic variables such as quality of life, nutritional intake, or treatment-related adverse events were not included, which may influence outcomes. Finally, all models were internally validated; multi-center prospective studies are needed to evaluate generalizability. Despite these limitations, this study is the first to demonstrate the synergistic value of pretreatment sarcopenia and non-contrast CT radiomics in prognostic prediction for sRCC, offering a novel non-invasive approach for preoperative risk stratification. The combined model exhibits not only high predictive accuracy but also clinical interpretability—SHAP analysis clarified the contribution of each feature, enhancing the credibility and potential clinical utility of the model.

## Conclusion

This study demonstrates that a combined model integrating preoperative sarcopenia and non-contrast CT-based radiomic features significantly improves the prediction of postoperative survival in patients with sRCC. The model outperformed radiomics-only predictions and showed robust discriminative ability and clinical utility. These findings support the use of sarcopenia and radiomics as complementary preoperative biomarkers for individualized prognostic assessment and treatment planning in this high-risk population.

## Data Availability

The original contributions presented in the study are included in the article/**Supplementary Material**. Further inquiries can be directed to the corresponding author/s.

## References

[1] B S Aj P F P DS F JW S AS B. Quality of pathological reporting for renal cell cancer: implications for systemic therapy, prognostication and surveillance. BJU Int. (2011) 108:343–8. doi: 10.1111/j.1464-410X.2010.09871.x, PMID: 21087450

[2] Shuch B Said J LaRochelle JC Zhou Y Li G Klatte T. Histologic evaluation of metastases in renal cell carcinoma with sarcomatoid transformation and its implications for systemic therapy. Cancer. (2010) 116:616–24. doi: 10.1002/cncr.24768, PMID: 19998348 PMC3162346

[3] Cheville JC Lohse CM Zincke H Weaver AL Leibovich BC Frank I. Sarcomatoid renal cell carcinoma: an examination of underlying histologic subtype and an analysis of associations with patient outcome. Am J Surg Pathol. (2004) 28:435–41. doi: 10.1097/00000478-200404000-00002, PMID: 15087662

[4] T K K Z-S J D HY L B Y M F. Using percentage of sarcomatoid differentiation as a prognostic factor in renal cell carcinoma. Clin Genitourin Cancer. (2015) 13:225–30. doi: 10.1016/j.clgc.2014.12.001, PMID: 25544725

[5] Delahunt B. Sarcomatoid renal carcinoma: the final common dedifferentiation pathway of renal epithelial Malignancies. Pathology. (1999) 31:185–90. doi: 10.1080/003130299104945, PMID: 10503259

[6] de Peralta-Venturina M Moch H Amin M Tamboli P Hailemariam S Mihatsch M. Sarcomatoid differentiation in renal cell carcinoma: a study of 101 cases. Am J Surg Pathol. (2001) 25:275–84. doi: 10.1097/00000478-200103000-00001, PMID: 11224597

[7] Mian BM Bhadkamkar N Slaton JW Pisters PW Daliani D Swanson DA. Prognostic factors and survival of patients with sarcomatoid renal cell carcinoma. J Urol. (2002) 167:65–70. doi: 10.1016/S0022-5347(05)65384-0 11743277

[8] Salgia NJ Aubrecht WM Wang L Ram B Wasik BJ Khan A. Stratification of patients with renal cell carcinoma by the abundance of sarcomatoid features reveals differences in survival and the underlying pathobiology. Eur Urol Oncol. (2024) 7:S258893112400052X. doi: 10.1016/j.euo.2024.02.007, PMID: 38480032 PMC11390971

[9] Wang Z Zeng X Chen R Chen Z. Ki-67 index and percentage of sarcomatoid differentiation were two independent prognostic predictors in sarcomatoid renal cell carcinoma. Cancer Manag Res. (2018) 10:5339–47. doi: 10.2147/CMAR.S176242, PMID: 30464630 PMC6225922

[10] Heng DYC Xie W Regan MM Warren MA Golshayan AR Sahi C. Prognostic factors for overall survival in patients with metastatic renal cell carcinoma treated with vascular endothelial growth factor-targeted agents: results from a large, multicenter study. J Clin Oncol. (2009) 27:5794–9. doi: 10.1200/JCO.2008.21.4809, PMID: 19826129

[11] Karam JA Uzzo R Bex A Leung W Tat C Nicholas A. Adjuvant atezolizumab in patients with sarcomatoid renal cell carcinoma: A prespecified subgroup analysis of IMmotion010. Eur Urol Oncol. (2024) 7:1175–8. doi: 10.1016/j.euo.2024.06.006, PMID: 38955577

[12] Wu C-H Liang P-C Hsu C-H Chang FT Shao YY Ting-Fang Shih T. Total skeletal, psoas and rectus abdominis muscle mass as prognostic factors for patients with advanced hepatocellular carcinoma. J Formos Med Assoc. (2021) 120:559–66. doi: 10.1016/j.jfma.2020.07.005, PMID: 32651043

[13] Huillard O Mir O Peyromaure M Tlemsani C Giroux J Boudou-Rouquette P. Sarcopenia and body mass index predict sunitinib-induced early dose-limiting toxicities in renal cancer patients. Br J Cancer. (2013) 108:1034–41. doi: 10.1038/bjc.2013.58, PMID: 23462722 PMC3619075

[14] Feliciano EMC Kroenke CH Meyerhardt JA Prado CM Bradshaw PT Kwan ML. Association of systemic inflammation and sarcopenia with survival in nonmetastatic colorectal cancer: results from the C SCANS study. JAMA Oncol. (2017) 3:e172319. doi: 10.1001/jamaoncol.2017.2319, PMID: 28796857 PMC5824285

[15] Pudjihartono N Fadason T Kempa-Liehr AW O'Sullivan JM. A review of feature selection methods for machine learning-based disease risk prediction. Front Bioinform. (2022) 2:927312. doi: 10.3389/fbinf.2022.927312, PMID: 36304293 PMC9580915

[16] Zeng S Wang X-L Yang H. Radiomics and radiogenomics: extracting more information from medical images for the diagnosis and prognostic prediction of ovarian cancer. Mil Med Res. (2024) 11:77. doi: 10.1186/s40779-024-00580-1, PMID: 39673071 PMC11645790

[17] La Greca Saint-Esteven A Vuong D Tschanz F van Timmeren JE Dal Bello R Waller V. Systematic review on the association of radiomics with tumor biological endpoints. Cancers (Basel). (2021) 13:3015. doi: 10.3390/cancers13123015, PMID: 34208595 PMC8234501

[18] Kocak B Durmaz ES Erdim C Ates E Kaya OK Kilickesmez O. Radiomics of renal masses: systematic review of reproducibility and validation strategies. AJR Am J Roentgenol. (2020) 214:129–36. doi: 10.2214/AJR.19.21709, PMID: 31613661

[19] Shu J Wen D Xi Y Xia Y Cai Z Xu W. Clear cell renal cell carcinoma: Machine learning-based computed tomography radiomics analysis for the prediction of WHO/ISUP grade. Eur J Radiol. (2019) 121:108738. doi: 10.1016/j.ejrad.2019.108738, PMID: 31756634

[20] Coy H Hsieh K Wu W Nagarajan MB Young JR Douek ML. Deep learning and radiomics: the utility of Google TensorFlow™ Inception in classifying clear cell renal cell carcinoma and oncocytoma on multiphasic CT. Abdom Radiol (NY). (2019) 44:2009–20. doi: 10.1007/s00261-019-01929-0, PMID: 30778739

[21] van Griethuysen JJM Fedorov A Parmar C Hosny A Aucoin N Narayan V. Computational radiomics system to decode the radiographic phenotype. Cancer Res. (2017) 77:e104–7. doi: 10.1158/0008-5472.CAN-17-0339, PMID: 29092951 PMC5672828

[22] Carey EJ Lai JC Wang CW Dasarathy S Lobach I Montano-Loza AJ. A multicenter study to define sarcopenia in patients with end-stage liver disease. Liver Transpl. (2017) 23:625–33. doi: 10.1002/lt.24750, PMID: 28240805 PMC5762612

[23] Prado CMM Lieffers JR McCargar LJ Reiman T Sawyer MB Martin L. Prevalence and clinical implications of sarcopenic obesity in patients with solid tumours of the respiratory and gastrointestinal tracts: a population-based study. Lancet Oncol. (2008) 9:629–35. doi: 10.1016/S1470-2045(08)70153-0, PMID: 18539529

[24] Hamaguchi Y Kaido T Okumura S Kobayashi A Hammad A Tamai Y. Proposal for new diagnostic criteria for low skeletal muscle mass based on computed tomography imaging in Asian adults. Nutrition. (2016) 32:1200–5. doi: 10.1016/j.nut.2016.04.003, PMID: 27292773

[25] Guichet PL Taslakian B Zhan C Aaltonen E Farquharson S Hickey R. MRI-derived sarcopenia associated with increased mortality following yttrium-90 radioembolization of hepatocellular carcinoma. Cardiovasc Intervent Radiol. (2021) 44:1561–9. doi: 10.1007/s00270-021-02874-6, PMID: 34089074

[26] Lundberg SM Erion G Chen H DeGrave A Prutkin JM Nair B. From local explanations to global understanding with explainable AI for trees. Nat Mach Intell. (2020) 2:56–67. doi: 10.1038/s42256-019-0138-9, PMID: 32607472 PMC7326367

[27] Nohara Y Matsumoto K Soejima H Nakashima N. Explanation of machine learning models using shapley additive explanation and application for real data in hospital. Comput Methods Programs BioMed. (2022) 214:106584. doi: 10.1016/j.cmpb.2021.106584, PMID: 34942412

[28] Khan AI Psutka SP Patil DH Hong G Williams MA Bilen MA. Sarcopenia and systemic inflammation are associated with decreased survival after cytoreductive nephrectomy for metastatic renal cell carcinoma. Cancer. (2022) 128:2073–84. doi: 10.1002/cncr.34174, PMID: 35285950

[29] Yang N Zhou P Lyu J Ren J Nie X Zhao S. Prognostic value of sarcopenia and myosteatosis alterations on survival outcomes for esophageal squamous cell carcinoma before and after radiotherapy. Nutrition. (2024) 127:112536. doi: 10.1016/j.nut.2024.112536, PMID: 39182329

[30] Liu Z Wu Y Khan AA Lun LU Wang J Chen J. Deep learning-based radiomics allows for a more accurate assessment of sarcopenia as a prognostic factor in hepatocellular carcinoma. J Zhejiang Univ Sci B. (2024) 25:83–90. doi: 10.1631/jzus.B2300363, PMID: 38163668 PMC10758209

[31] Li X Ding P Wu J Wu H Yang P Guo H. Preoperative sarcopenia and postoperative accelerated muscle loss negatively impact survival after resection of locally advanced gastric cancer. BMC Cancer. (2025) 25:269. doi: 10.1186/s12885-025-13674-3, PMID: 39953409 PMC11829415

[32] Mo X Shen L Cheng R Wang P Wen L Sun Y. Faecal microbiota transplantation from young rats attenuates age-related sarcopenia revealed by multiomics analysis. J Cachexia Sarcopenia Muscle. (2023) 14:2168–83. doi: 10.1002/jcsm.13294, PMID: 37439281 PMC10570072

[33] Nardone OM de Sire R Petito V Testa A Villani G Scaldaferri F. Inflammatory bowel diseases and sarcopenia: the role of inflammation and gut microbiota in the development of muscle failure. Front Immunol. (2021) 12:694217. doi: 10.3389/fimmu.2021.694217, PMID: 34326845 PMC8313891

[34] Arcaro A Lepore A Cetrangolo GP Paventi G Ames PRJ Gentile F. A reassessment of sarcopenia from a redox perspective as a basis for preventive and therapeutic interventions. Int J Mol Sci. (2025) 26:7787. doi: 10.3390/ijms26167787, PMID: 40869107 PMC12386734

[35] Salazar N Arboleya S Fernández-Navarro T de Los Reyes-Gavilán CG Gonzalez S Gueimonde M. Age-associated changes in gut microbiota and dietary components related with the immune system in adulthood and old age: A cross-sectional study. Nutrients. (2019) 11:1765. doi: 10.3390/nu11081765, PMID: 31370376 PMC6722604

[36] Santoro A Ostan R Candela M Biagi E Brigidi P Capri M. Gut microbiota changes in the extreme decades of human life: a focus on centenarians. Cell Mol Life Sci. (2018) 75:129–48. doi: 10.1007/s00018-017-2674-y, PMID: 29032502 PMC5752746

[37] Rampelli S Soverini M D’Amico F Barone M Tavella T Monti D. Shotgun metagenomics of gut microbiota in humans with up to extreme longevity and the increasing role of xenobiotic degradation. mSystems. (2020) 5:e00124–20. doi: 10.1128/mSystems.00124-20, PMID: 32209716 PMC7093822

[38] Han D-S Wu W-K Liu P-Y Yang Y-T Hsu H-C Kuo C-H. Differences in the gut microbiome and reduced fecal butyrate in elders with low skeletal muscle mass. Clin Nutr. (2022) 41:1491–500. doi: 10.1016/j.clnu.2022.05.008, PMID: 35667265

[39] Zhang L Liao J Chen Q Chen M Kuang Y Chen L. Characterization of the gut microbiota in frail elderly patients. Aging Clin Exp Res. (2020) 32:2001–11. doi: 10.1007/s40520-019-01385-2, PMID: 31656031

[40] McNabney SM Henagan TM. Short chain fatty acids in the colon and peripheral tissues: A focus on butyrate, colon cancer, obesity and insulin resistance. Nutrients. (2017) 9:1348. doi: 10.3390/nu9121348, PMID: 29231905 PMC5748798

[41] Thevaranjan N Puchta A Schulz C Naidoo A Szamosi JC Verschoor CP. Age-associated microbial dysbiosis promotes intestinal permeability, systemic inflammation, and macrophage dysfunction. Cell Host Microbe. (2018) 23:570. doi: 10.1016/j.chom.2018.03.006, PMID: 29649447 PMC5899819

[42] Cheng Y Lin S Cao Z Yu R Fan Y Chen J. The role of chronic low-grade inflammation in the development of sarcopenia: Advances in molecular mechanisms. Int Immunopharmacol. (2025) 147:114056. doi: 10.1016/j.intimp.2025.114056, PMID: 39799736

[43] Xiang Y Dai J Xu L Li X Jiang J Xu J. Research progress in immune microenvironment regulation of muscle atrophy induced by peripheral nerve injury. Life Sci. (2021) 287:120117. doi: 10.1016/j.lfs.2021.120117, PMID: 34740577

[44] Fried LP Tangen CM Walston J Newman AB Hirsch C Gottdiener J. Frailty in older adults: evidence for a phenotype. J Gerontol A Biol Sci Med Sci. (2001) 56:M146–156. doi: 10.1093/gerona/56.3.m146, PMID: 11253156

[45] Shafiee G Ostovar A Heshmat R Darabi H Sharifi F Raeisi A. Bushehr Elderly Health (BEH) programme: study protocol and design of musculoskeletal system and cognitive function (stage II). BMJ Open. (2017) 7:e013606. doi: 10.1136/bmjopen-2016-013606, PMID: 28780537 PMC5577871

[46] Rolland Y Czerwinski S Abellan Van Kan G Morley JE Cesari M Onder G. Sarcopenia: its assessment, etiology, pathogenesis, consequences and future perspectives. J Nutr Health Aging. (2008) 12:433–50. doi: 10.1007/BF02982704, PMID: 18615225 PMC3988678

[47] Jung HN Jung CH Hwang Y-C. Sarcopenia in youth. Metabolism. (2023) 144:155557. doi: 10.1016/j.metabol.2023.155557, PMID: 37080353

[48] Weng Y-S Tang C-T Chang W-C Huang GS Chiu CH Chiang SW. Added value of 18Fluorine-fluorodeoxyglucose (18F-FDG) PET/MRI for evaluation of failed back surgery syndrome: comparison with non-contrast MRI. Jpn J Radiol. (2025) 43:509–19. doi: 10.1007/s11604-024-01679-0, PMID: 39404924

[49] Xi IL Zhao Y Wang R Chang M Purkayastha S Chang K. Deep learning to distinguish benign from Malignant renal lesions based on routine MR imaging. Clin Cancer Res. (2020) 26:1944–52. doi: 10.1158/1078-0432.CCR-19-0374, PMID: 31937619

[50] Choi JW Hu R Zhao Y Purkayastha S Wu J McGirr AJ. Preoperative prediction of the stage, size, grade, and necrosis score in clear cell renal cell carcinoma using MRI-based radiomics. Abdom Radiol (NY). (2021) 46:2656–64. doi: 10.1007/s00261-020-02876-x, PMID: 33386910 PMC11193204

[51] Zhao J Xu H Fu Y Ding X Wang M Peng C. Development and validation of intravoxel incoherent motion diffusion weighted imaging-based model for preoperative distinguishing nuclear grade and survival of clear cell renal cell carcinoma complicated with venous tumor thrombus. Cancer Imaging. (2024) 24:164. doi: 10.1186/s40644-024-00816-2, PMID: 39695867 PMC11654007

[52] Frank I Blute ML Cheville JC Lohse CM Weaver AL Zincke H. An outcome prediction model for patients with clear cell renal cell carcinoma treated with radical nephrectomy based on tumor stage, size, grade and necrosis: the SSIGN score. J Urol. (2002) 168:2395–400. doi: 10.1016/S0022-5347(05)64153-5, PMID: 12441925

[53] Leibovich BC Blute ML Cheville JC Lohse CM Frank I Kwon ED. Prediction of progression after radical nephrectomy for patients with clear cell renal cell carcinoma: a stratification tool for prospective clinical trials. Cancer. (2003) 97:1663–71. doi: 10.1002/cncr.11234, PMID: 12655523

[54] Li W Yang T Li P Liu P Zhang S Zhu J. Multicenter evaluation of CT deep radiomics model in predicting Leibovich score risk groups for non-metastatic clear cell renal cell carcinoma. Displays. (2024) 85:102867. doi: 10.1016/j.displa.2024.102867

