# Supplementary material for: Integrating sarcopenia and non-contrast CT radiomics for preoperative prediction of survival in sarcomatoid renal cell carcinoma
Source: Front Oncol. 2025 Oct 22;15:1637032. doi: 10.3389/fonc.2025.1637032 (PMC12585953; doi:10.3389/fonc.2025.1637032)
Supplement: Supplementary file 1 [file SupplementaryFile1.docx]

*
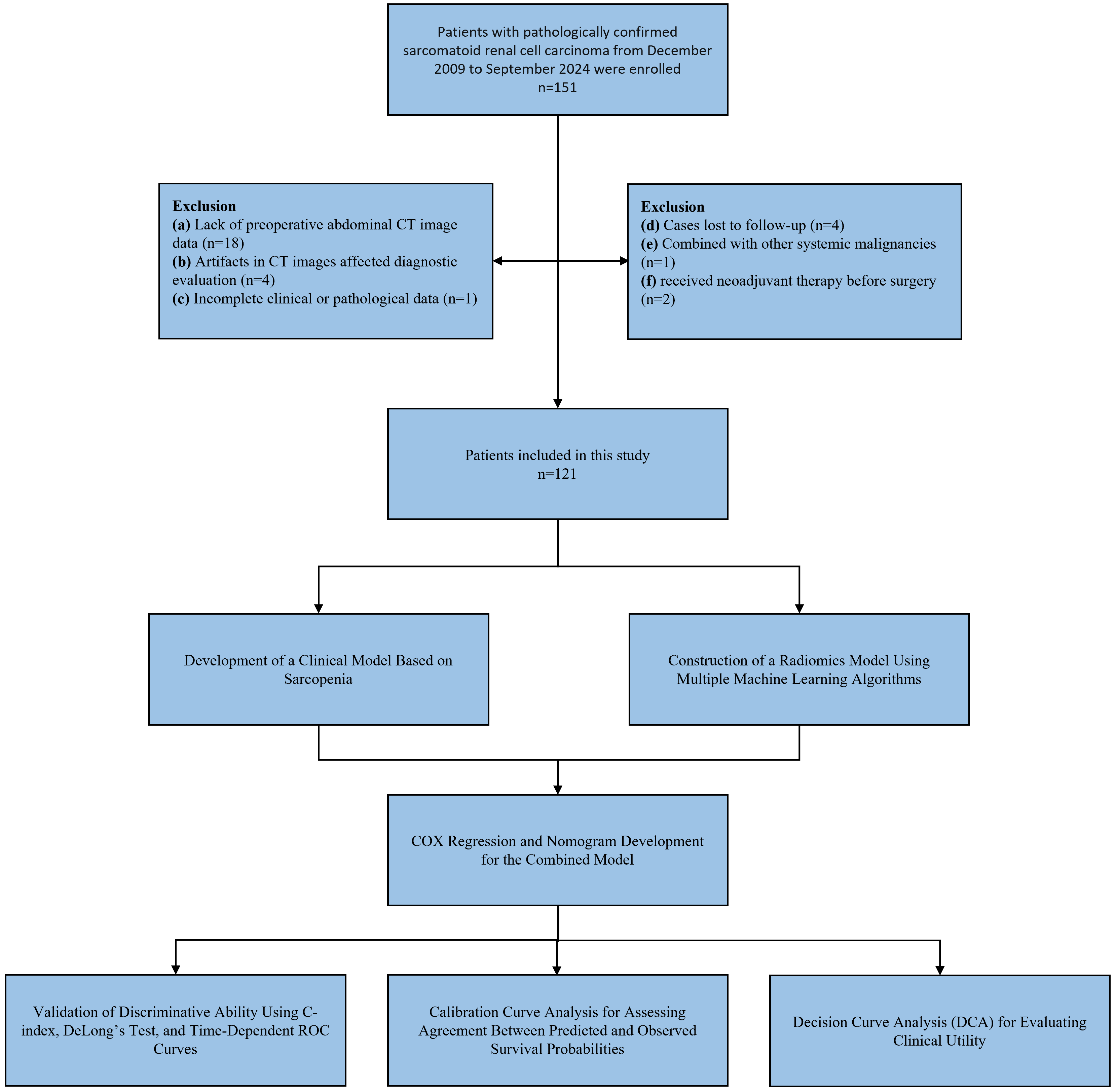
*

*Supplementary Figure 1. Study Flowchart: Patient Selection, Model Development, and Validation Process for Predicting Outcomes in Sarcomatoid Renal Cell Carcinoma Based on Clinical and Radiomics Features*

*
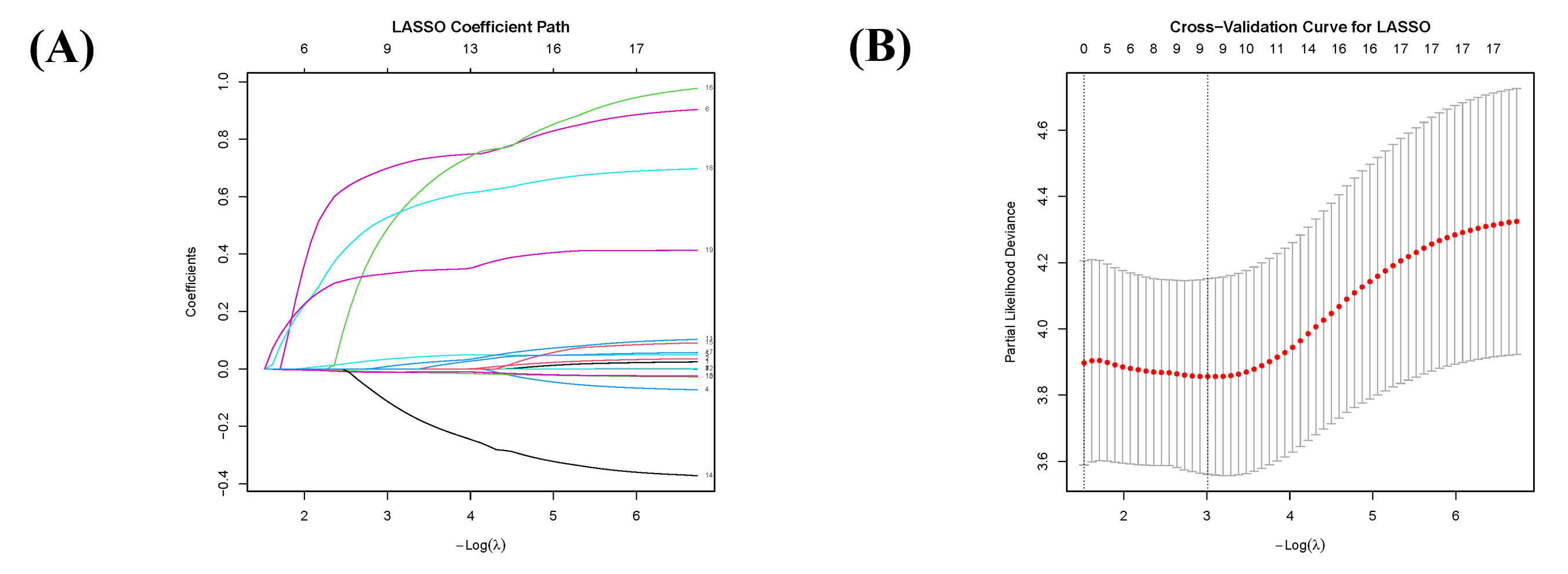
*

*Supplementary Figure 2. Variable selection using the LASSO Cox regression model for the clinical predictor set. (A) Coefficient profiles of candidate clinical variables associated with overall survival in sarcomatoid renal cell carcinoma (sRCC) across different LASSO penalty (λ) values. (B) Ten-fold cross-validation for optimal lambda (λ) selection. The dotted vertical lines indicate the minimum criteria (left) and one standard error (SE) criteria (right).*
